# Factor V Leiden, estrogen, and multimorbidity association with venous thromboembolism in a British-South Asian cohort

**DOI:** 10.1016/j.isci.2023.107795

**Published:** 2023-09-01

**Authors:** Emma F. Magavern, Genes & Health Research Team, Damian Smedley, Mark J. Caulfield

**Affiliations:** 1 William Harvey Research Institute, Queen Mary University of London, London EC1M 6BQ, UK; 2 Genes & Health, Blizard Institute, Queen Mary University of London, London E1 2AB, UK

**Keywords:** Cardiovascular medicine, Public health, Clinical endocrinology

## Abstract

Multimorbidity, estrogen use, and Factor V Leiden (FVL) are known independent risk factors for venous thromboembolism (VTE). This cross-sectional analysis of women in the Genes & Health British-South Asian cohort (N 20,048) linked the *F5* SNP rs6025 with estrogen prescribing data and VTE events. Multivariable logistic regression was used to test the association between estrogen use, FVL, common medical co-morbidities, and VTE. Estrogens were prescribed to 30% of women. 3% of participants were FVL carriers. 439 participants had a VTE event (2.2%), and VTE prevalence increased with obesity, hypertension, dyslipidemia, chronic kidney disease, estrogen use, and in the presence of FVL. One medical condition above was independently associated with VTE with an OR 1.6 (CI 1.2–2.0, p 0.001); two medical conditions OR 2.7 (CI 2.0–3.7, p < 0.001); three OR 5.3 (CI 3.8–7.4, p < 0.001); four OR 8.1 (CI 4.9–13.0, p < 0.001). Multimorbidity and FVL compound risk of VTE with estrogen use.

## Highlights

• Exogenous estrogens are commonly prescribed to women
• Multimorbidity and FVL compound risk of VTE with estrogen use
• FVL is an important contributor to VTE with increasing medical multimorbidity
• This is important for women in deprived populations with high multimorbidity burden

## Graphical abstract

**Figure:**
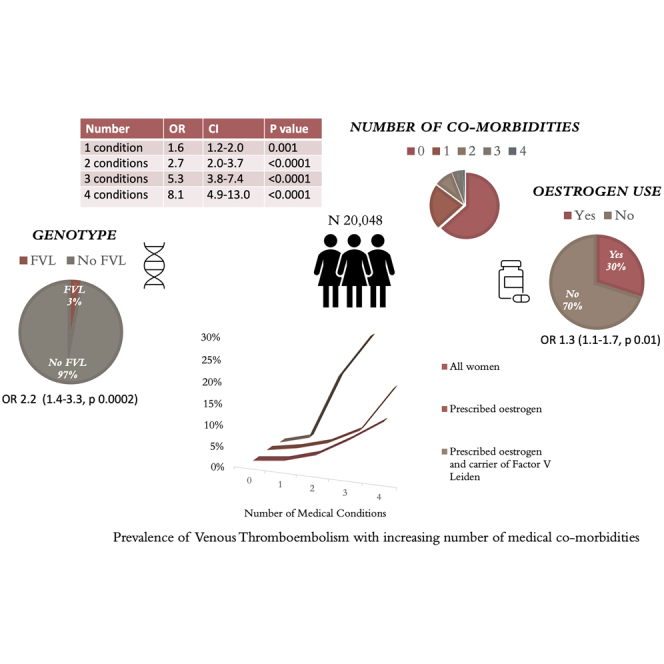

## Teaser

Cardiovascular medicine; Public health; Reproductive health

## Introduction

Incidence of venous thromboembolism (VTE) varies by age and ethnicity, with estimates ranging from 104 to 183 events per 100,000 person-years in European ancestry populations.^1^ There is some evidence of lower incidence in East Asian populations.^1^ VTE results in significant morbidity and mortality, contributing to impaired quality of life and high health care costs.^2^^,^^3^^,^^4^ Mortality from pulmonary embolism is significant and noted recently to be up-trending among younger patients (25–64 years old) in the USA.^5^^,^^6^

Estrogen containing oral combined contraception (OCP) and hormone replacement therapy (HRT) are commonly used by pre- and post-menopausal women. Data from the USA suggests that more than 80% of sexually active women had taken oral contraceptives, almost all of which were combination therapies containing estrogen.^7^^,^^8^ OCPs and Estrogen containing HRT are known to increase the relative risk of venous thromboembolism (VTE) significantly, though the absolute risk of VTE remains low.^9^^,^^10^

Factor V Leiden (FVL) is caused by a single nucleotide polymorphism in the *F5* gene (1691G>A substitution) and leads to a prothrombotic state, which has a synergistic increase in VTE risk with exogenous estrogen use.^11^^,^^12^ The mechanism is a resistance to activated protein C, which is an endogenous anticoagulant.^11^ Prevalence of FVL is known to vary across *trans*-ancestral groups, with highest prevalence in European ancestry and a lower prevalence in Asian populations.^13^^,^^14^

Polymorbidity is increasing, and it remains unclear how intersection of multiple common medical conditions, exogenous estrogen use, and FVL may alter risk of VTE.^15^ To address this gap in knowledge we analyzed the Genes & Health (G&H) cohort of Bangladeshi and Pakistani ancestry participants in the United Kingdom (UK). Although Asian populations are known to have lower prevalence of the FVL allele, as compared with European ancestry populations, the G&H cohort suffers from high rates of cardio-metabolic morbidity and a large percentage of women are likely to be exposed to exogenous estrogen across their lifetime. It is also a population that is grossly under-represented in clinical and preclinical research cohorts.

Pharmacogenomic panels are being considered for routine use in national clinical care in the United Kingdom’s National Health Service, therefore revisiting utility of *F5* pharmacogenomic testing to inform choice of contraception and HRT is timely.^16^ Pharmacogenomic panel testing shifts the issue of FVL testing from a population screening question and reframes it as a medicine optimization tool. Furthermore, prior health economic models used to estimate cost of genetic testing are obsolete in this context.

The aim of this study is to clarify how intersection of multiple common medical conditions, exogenous estrogen use, and FVL contribute to cumulative risk of VTE in a British South-Asian ancestry cohort.

## Results

### *F5* genotype and exogenous estrogen use in the G&H cohort

In this study cohort 2.8% of women had at least 1 copy of the FVL polymorphism, and 30% had been prescribed estrogens (Table 1). In the sub cohort of women prescribed estrogens 2.6% had an FVL polymorphism (Table 1).

**Table 1:** Percentage of G&H cohort with 1 *F5* Leiden mutation (heterozygotes) and 2 *F5* Leiden mutations (homozygotes)

Number of *F5* Leiden mutations	1 Heterozygous	2 Homozygous	All *F5* Leiden carriers
All Women (N 20,048)	2.7% (547)	0.05% (11)	2.8% (558)
Women prescribed estrogen (N 5,970)	2.5% (152)	0% (1)	2.6% (153)

### VTE events

The relative risk of VTE in women carrying a FVL mutation who had been prescribed estrogen was more than double women who did not have a FVL mutation (4.6% vs. 2.1%, significant on fisher’s exact testing p 0.047, OR 2.2, 95% CI 0.9–4.9). The majority of the 439 VTE events were phlebitis and thrombophlebitis (76%). Of those women prescribed estrogens, 21% of the participants with VTE (27/129) had a pulmonary embolism (0.5% of all women prescribed estrogen) (Table 2).

**Table 2:** Female cohort characteristics and VTE events

Characteristics and co-morbid conditions	Prevalence in all Women (N 20,048)	Prevalence in Women prescribed estrogens (N 5,970)	Prevalence in Women not prescribed estrogens (N 14,078)	p value
Diabetes mellitus	15% (3,068)	11% (639)	17% (2,429)	<0.0001∗∗
Obesity	22% (4,410)	21% (1,231)	23% (3,179)	0.002∗
Primary Hypertension	16% (3,298)	11% (628)	19% (2,670)	<0.0001∗∗
Dyslipidemia	16% (3,192)	11% (627)	18% (2,565)	<0.0001∗∗
Chronic Kidney Disease	4% (831)	2% (111)	5% (720)	<0.0001∗∗
Mean Age at enrollment (years)	39 years old (+/− 13.2)	37 years old (+/− 9.6)	40 years old (+/− 14.3)	<0.0001∗∗

**VTE events**				

Pulmonary embolism	0.5% (96)	0.5% (27)	0.5% (69)	0.8
Phlebitis and thrombophlebitis	1.7% (340)	1.8% (108)	1.6% (232)	0.4
Other venous embolism and thrombosis	0.1% (23)	0% (0)	0.2% (23)	0.0004∗∗
Portal vein thrombosis	0.05% (10)	0% (1)	0.1% (9)	0.3
Total number of participants with VTE	2.2% (439)	2.2% (129)	2.2% (310)	0.9
FVL (homozygous or heterozygous)	2.7% (558)	2.6% (153)	2.9% (405)	0.22

p values from fisher’s exact test for discrete variables and t-test for continuous variables. ∗p value < 0.05, ∗∗p value < 0.001.

### Multimorbidity

Prevalence of common medical comorbidities in the cohort prescribed estrogens are shown in Table 2. Those who had been prescribed estrogens were young at enrollment (mean age 37), with 21% obesity, 11% diabetes mellitus, 11% primary hypertension (HTN), 11% dyslipidemia, and 2% chronic kidney disease (CKD). Those prescribed estrogens were significantly younger and less likely to have a diagnosis of obesity, diabetes mellitus, primary hypertension, dyslipidemia, or chronic kidney disease as compared with the cohort who had not been prescribed estrogens. However, there was no significant difference in FVL prevalence between the two groups.

### Association of FVL, estrogen, and multimorbidity with VTE events

Estrogen use was independently associated with VTE (OR 1.3, CI 1.1–1.7, p value 0.009), as was FVL carrier status (OR 2.2, 1.4–3.3, p 0.001), and age (OR 1.01 (per year), 1.01–1.02 p 0.002) (Table 3). Obesity (OR 1.5, CI 1.2–1.9, p 0.001), HTN (OR 1.8, CI 1.3–2.4, p < 0.001), dyslipidemia (OR 1.7, CI 1.3–2.2, p 0.002), and CKD (OR 2.0, CI 1.5–2.7, p < 0.001) were also associated independently with higher VTE prevalence (Table 3).

**Table 3:** Multivariable logistic regression in female cohort (**N 20,048**) examining associations with VTE

Exposure	OR for VTE	95% CI	p value
FVL carrier	2.2	1.4–3.3	0.0002∗∗
Estrogen use	1.3	1.1–1.7	0.009∗
Obesity	1.5	1.2–1.9	0.0001∗∗
Hypertension	1.8	1.3–2.4	<0.0001∗∗
Dyslipidemia	1.7	1.3–2.2	0.0002∗∗
Chronic Kidney Disease	2.0	1.5–2.7	<0.0001∗∗
Diabetes mellitus	1.0	0.8–1.4	0.86
Age at enrollment	1.01 (per year)	1.01–1.02	0.002∗

∗p value < 0.05, ∗∗p value < 0.001.

Diagnosis of any one medical condition including obesity, dyslipidemia, HTN, and CKD was independently associated with VTE with an OR 1.6 (1.2–2.0, p 0.001); two co-occurring medical conditions OR 2.7 (2.0–3.7, p < 0.001); three co-occurring conditions OR 5.3 (3.8–7.4, p < 0.001); four co-occurring conditions OR 8.1 (4.9–13.0, p < 001) (Table 4).

**Table 4:** Multivariable logistic regression testing for association between VTE and multiple of the 4 common medical conditions found to be significant above (obesity, HTN, CKD, dyslipidemia)

Exposure	OR for VTE	95% CI	p value
FVL Leiden carrier	2.2	1.4–3.3	0.0002∗∗
Estrogen use	1.3	1.1–1.7	0.01∗

**Poly-morbidity: (Obesity, Dyslipidemia, Hypertension, Chronic kidney disease)**			

1 condition	1.6	1.2–2.0	0.001∗
2 conditions	2.7	2.0–3.7	<0.0001∗∗
3 conditions	5.3	3.8–7.4	<0.0001∗∗
4 conditions	8.1	4.9–13.0	<0.0001∗∗
Age at enrollment	1.02 (per year)	1.01–1.03	0.0001∗∗

Odds ratio (OR), Confidence interval (CI).

20 principal components were included as covariates to control for population stratification.

∗p value < 0.05, ∗∗p value < 0.001.

When VTE events were stratified by poly-morbidity status, prevalence of VTE increases with number of conditions (Table 5). Our results show that the absolute risk of VTE in this cohort is not trivial in those women with two or more co-existent medical conditions in the absence of Estrogen use, ∼4% with two conditions, rising to ∼14% with four co-morbid conditions.

**Table 5:** Multimorbidity impact

Number of at risk common medical co-morbidities	0	1	2	3	4
**Women** (N 20,048)	63% (12688)	22% (4386)	9% (1799)	5% (953)	1% (222)
**VTE prevalence in Women**	**1.2%** (157/12688)	**2.1%∗∗** (92/4386)	**4.3%∗∗** (78/1799)	**8.6%∗∗** (82/953)	**13.5%∗** (30/222)
**Women prescribed Estrogen** (N 5970)	68.5% (4088)	22.4% (1337)	6.6% (396)	2.1% (128)	0.4% (21)
**VTE prevalence in Women prescribed Estrogen**	**1.4%** (59/4088)	**2.7%∗** (36/1337)	**4.8%∗** (19/396)	**8.6%** (11/128)	**19.0%** (4/21)
**Women prescribed Estrogen in presence of Factor V Leiden** (N 153)	63% (97)	22% (33)	10% (16)	5% (7)	0% (0)
**VTE prevalence in Women prescribed Estrogen in presence of Factor V Leiden**	**1%** (1/97)	**3%** (1/33)	**19%** (3/16)	**29%** (2/7)	NA

The above table outlines VTE prevalence in participants with increasing numbers of 4 common medical co-morbidities associated with VTE in this cohort: Obesity, hypertension, dyslipidemia, chronic kidney disease. Statistically significant difference with p value < 0.05 by Fisher’s exact test in comparison with the column to the left noted by ∗. ∗∗ denotes p value < 0.001.

**Figure 1:**
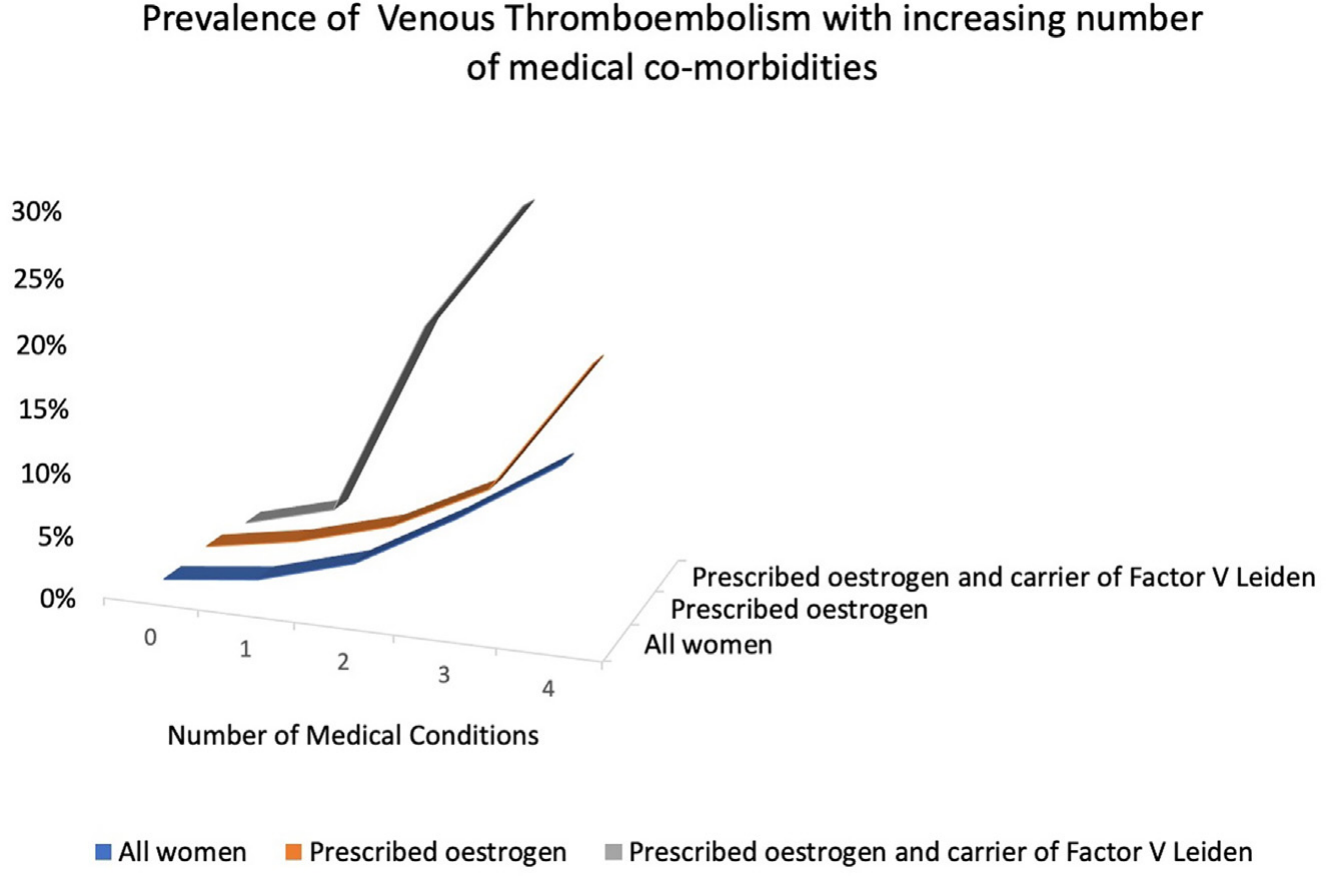
Prevalence of VTE Prevalence of VTE in women increases with increasing number of co-existent medical comorbidities identified on the x axis (obesity, hypertension, chronic kidney disease, dyslipidemia). There is an increase in VTE in those women prescribed estrogens that follows the same trend, increasing with number of medical co-morbidities. In those prescribed estrogen and carrying a Factor V Leiden mutation there is a steep increase in VTE risk in those women with more than one medical co-morbidity.

This risk is amplified with the same trends in those proscribed estrogens, though a smaller percentage of participants with multiple co-morbidities were prescribed estrogen compared with the baseline population (Table 5). In the sub-cohort prescribed estrogens who carry an FVL mutation there was an increase in VTE prevalence affecting those with more than one medical co-morbidity disproportionately (Table 5; Figure 1). 19% of those carrying an FVL mutation with two medical co-morbidities had a VTE event (compared with ∼5% VTE prevalence in those prescribed estrogens and having 2 medical co-morbidities overall) (Table 5). Likewise, in a dose-dependent fashion, those with three medical co-morbidities who carried an FVL mutation had a 29% prevalence of VTE (compared with ∼9% in those with 3 medical co-morbidities prescribed Estrogen overall) (Table 5; Figure 1).

## Discussion

**Figure 2:**
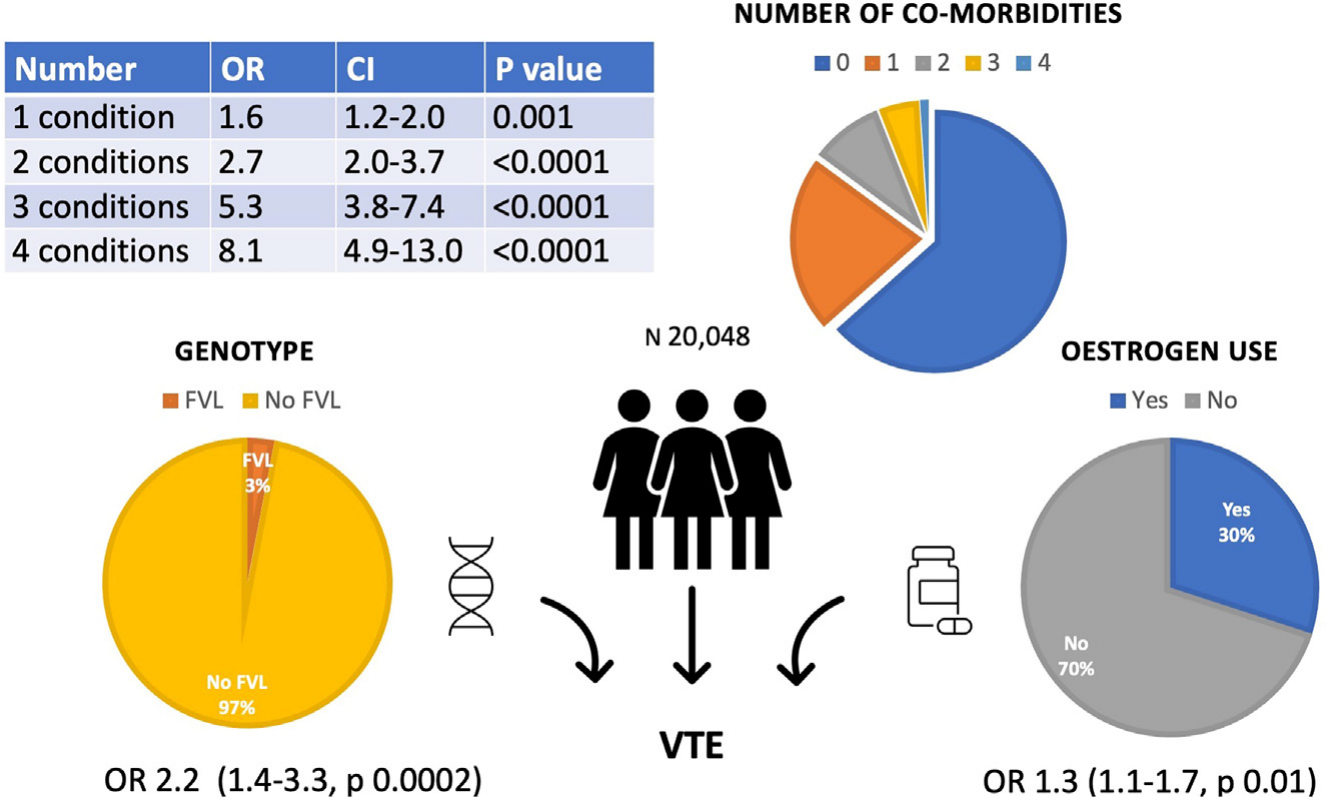
Study results overview 20, 048 UK women of Pakistani and Bangladeshi ancestry were analysed. Multivariable logistic regression was used to find the associations between estrogen use, FVL, common medical co-morbidities, and VTE. Estrogens were prescribed to 30% of women. 3% of participants were FVL carriers. VTE prevalence increased independently with obesity, hypertension, dyslipidemia, chronic kidney disease, estrogen use, and in the presence of FVL. Multiple of the common medical co-morbidities above (obesity, hypertension, dyslipidemia, chronic kidney disease) resulted in escalating risk of VTE independent of FVL or oestrogen use. Multimorbidity and FVL compound risk of VTE with estrogen use. OR, odds ratio, CI, confidence interval.

Our study shows an independent, statistically significant, and clinically meaningful increase in VTE prevalence in women who have FVL, had been prescribed estrogen, or had a diagnosis of obesity, HTN, CKD, or dyslipidemia. We demonstrated a cumulative significant association with VTE where several of these medical co-morbidities was present in combination, ranging from an OR 1.6 for one condition (CI 1.2–20, p 0.001) to OR 8.1 (CI 4.9–13.0, p < 0.001) for a participant with all four identified medical co-morbidities (not an uncommon patient to encounter in clinical practice) (Figure 2). This is the first such study to look at cumulative risk of common medical conditions, estrogen use and FVL on VTE prevalence in a South Asian ancestry western population. While independently these factors have all been associated with VTE to various degrees, prior studies have not aggregated commonly co-occurring medical conditions. Furthermore, South Asian ancestry populations in western countries are known to suffer from high rates of cardiometabolic morbidity.^17^

Our results show that the prevalence of VTE in this cohort is not trivial in those women with two or more co-existent medical conditions; rising to almost 1 in every 6 women with four comorbid conditions in the absence of Estrogen use. In the sub-cohort who had been prescribed estrogen the prevalence of VTE was nearly 1 in 5 for those women with all four medical conditions. In the presence of FVL, the prevalence of VTE with three medical conditions was nearly 1 in every 3 women. These absolute risks argue against prior dogma which resulted in a decision not to offer testing for FVL prior to estrogen prescription.^9^

Nearly 1 in every 3 women in this study cohort had been prescribed estrogens. The high prevalence of exposure to estrogens emphasizes the importance of elucidating multifactorial VTE risk.

VTE risk is known to be multifactorial, with inherited, acquired, and environmental risk factors. However, the contribution of multimorbidity with chronic and commonly overlapping cardiovascular and metabolic conditions to VTE has not been well studied. It is important to elucidate the cumulative impact of multimorbidity with exogenous estrogen use and FVL to optimize informed choice of estrogen containing medication use. Future studies should explore the impact of overlap in multimorbidity, FVL, and Estrogen use in other geographic, socioeconomic and ancestral populations. Further work should be done to understand the various aspects of multi morbidity that may be contributing to VTE risk such as lifestyle habits and environmental exposures associated with the studied medical conditions.

**Figure 3:**
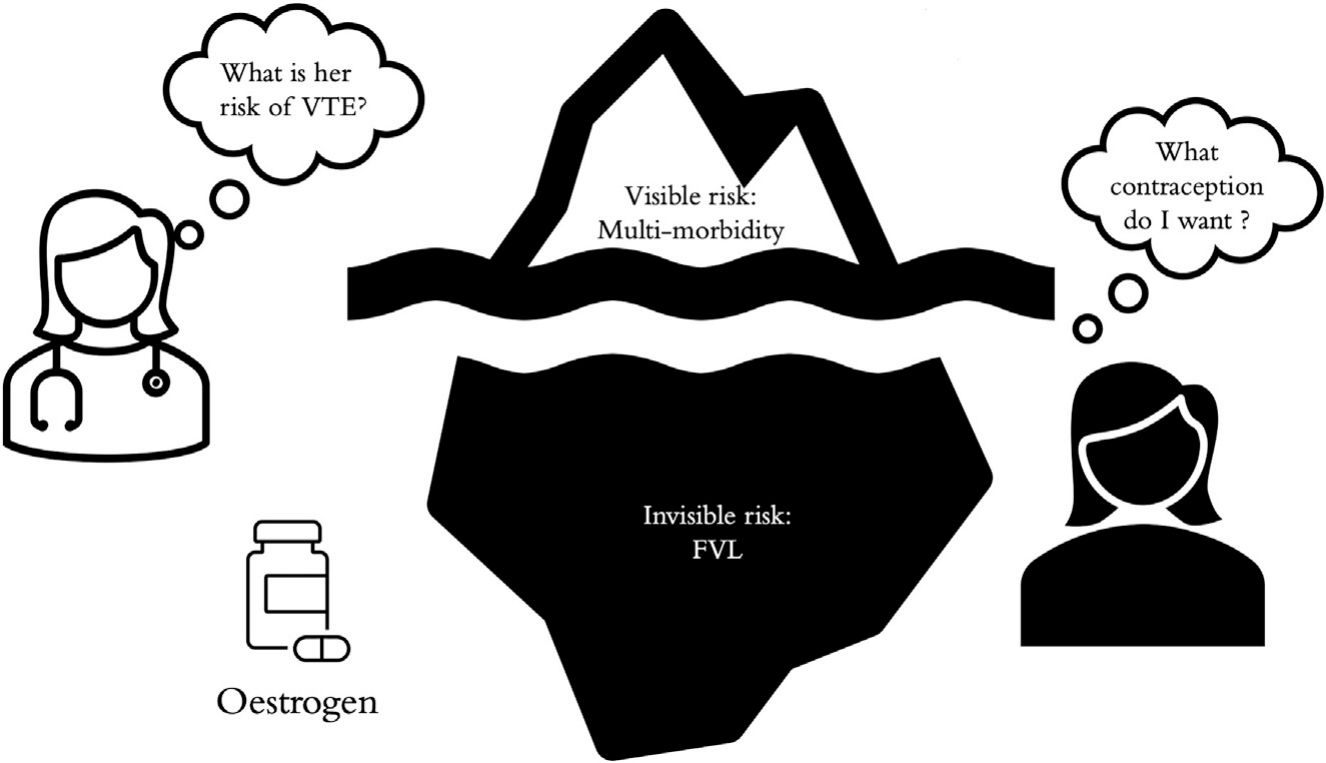
Clinical implications: as populations become increasingly multimorbid the invisible genetic risk of VTE posed by FVL is more relevant

Prior concerns were raised about women being denied contraception due to detection of FVL.^9^ However, there are many safe and effective non-estrogen containing choices for contraception and an estimation of non-trivial VTE risk does not need to be a contraindication to use. In fact, there is an increasing emphasis on wholistic decisions making rather than treating all thrombophilia as contraindications to OCP use.^18^ *F5* inclusion in a pharmacogenomic panel medicines optimization approach could therefore enable more personalized risk assessment and enable patients to make more informed decisions (Figure 3).

The population morbidity landscape has changed in the past several decades, as have contraception options and doctor patient decision making models. People are living longer but with more of life lived with co-morbidities.^19^ Projections suggest this will continue, with individuals experiencing four or more diseases estimated to reach 17% by 2035.^20^ Women are having children later in life and are more likely to have medical comorbidities during reproductive years than in the past.^21^^,^^22^ Contraception options have expanded, and there is now more emphasis on shared decision making and informed consent.

The healthcare provider landscape is shifting toward pre-emptive pharmacogenomic testing for commonly used non-specialist prescribed medications: The PREPARE trial demonstrated reduction of ADRs by 30% using a panel approach in European centers. In the UK, point of care genomic testing for *mtRNR1* has been initiated prior to aminoglycoside use in neonatal sepsis, and NICE is expected to shortly release guidelines recommending *CYP2C19* testing to guide choice of therapeutics after stoke.^23^^,^^24^^,^^25^ Therefore, a future when pre-emptive pharmacogenomic panels are readily available in routine care may be near at hand.

These factors combined mean that a large number of women who are likely to consider taking exogenous estrogens in their lifetime may have an elevated baseline risk of VTE due to co-morbidities and other multifactorial additional risk factors, and that pharmacogenomic panel information may be available to help inform personalized discussion of VTE risk. Including *F5* in such a panel would continue the shifting ideology of medicines optimization and shared decision making based on informed consent.

### Limitations of the study

Due to the overall low prevalence of VTE and of FVL, the number of women who had an FVL allele, had been prescribed estrogen, and had a VTE event was small (7 women). Therefore, it would be ideal to replicate these data in a larger cohort. Furthermore, due to limitations of the data available, this is a cross-sectional study. This approach and lack of longitudinal data are likely to decrease our signal and mean that we are underestimating the effect of estrogen on VTE and accounts for the lower OR associated with VTE from estrogen use in our study versus prior studies. However, this biases our model against signal detection, it does not compromise the validity of the significant associations we have presented. Furthermore, though the medical conditions considered could plausibly lead to increased VTE risk, there is not a plausible pathway for VTE to lead to the occurrence of these medical comorbidities. We also did not analyze drug-drug interactions.

### Clinical implications

As multimorbidity increases, it is important to examine cumulative risk of VTE from multiple common medical conditions, aging, and genetic risk prior to prescribing estrogen. FVL disproportionately increases VTE risk for those with multiple common medical co-morbidities taking estrogen contained in oral contraceptives due to additive risk. If these results are validated in other cohorts, it would suggest that not only obesity and HTN, but also dyslipidemia and chronic kidney disease should be considered and possibly even screened for before initiating estrogen therapy. Our cohort data suggests that clinicians are already less likely to prescribe estrogens to multimorbid patients, but that they are not less likely to prescribe estrogens to those with FVL (as it is not clinical practice to test for FVL in the absence of an unexplained thrombotic event or family history). This suggests the practice of asking about family history of VTE prior to prescribing estrogen is not significantly decreasing the percentage of patients with FVL being prescribed estrogens.

### Conclusions

FVL should be part of a pharmacogenomic panel to support medicine optimization as one factor in wholistic patient centered decision making regarding exogenous estrogen use. Even in lower prevalence genetic populations, FVL may be an important contributor to VTE in the context of increasing medical multimorbidity and high population level usage of estrogens. This is likely to be particularly important in deprived populations, as evidence suggests deprived people are disproportionately likely to be multimorbid.^26^

## STAR★Methods

### Key resources table

**Table:** 

REAGENT or RESOURCE	SOURCE	IDENTIFIER
**Software and algorithms**		

R studio	RStudio Team. RStudio: integrated development environment for R. Boston, MA: PBC; 2022. https://www.r-project.org/.	
PLINK	Purcell S, Chang C. PLINK 2.0. www.cog-genomics.org/plink/2.0/.	
Genes & Health Phenotypes	Genes & Health. Genes & Health: GeneAndHealth_PHENOTYPES. 2022. https://docs.google.com/spreadsheets/d/1ipwdF2j_owfr_QbkDYk1rk0TW3KtdfQYVQn-Vf-o38s/edit#gid=1517436704.	

### Resource availability

#### Lead contact

Further information should be directed to and will be fulfilled by the lead contact, Emma Magavern (e.magavern@qmul.ac.uk)

#### Materials availability

This study did not generate new unique reagents.

### Method details

#### The Genes &Health cohort

The Genes & Health (G&H) cohort study characteristics have been previously published.^27^ The cohort is comprised of Bangladeshi and Pakistani ancestry participants in the UK recruited from East London, Bradford and Manchester. G&H data access for this study was approved by the study executive committee. The London South East NRES Committee of the Health Research Authority has approved the G&H study, 14/LO/1240 (16 September 2014). Participants consented to electronic health record linkage and donated a saliva sample for DNA extraction.^27^ Cohort participants were genotyped using the Illumina GSAMD-24v3-0-EA chip.^28^

#### Characterization of F5 genotype in the G&H cohort

The *F5* SNP RS6025 (defining the presence of FVL) was genotyped on the chip as above and was extracted using PLINK 2.0.^29^^,^^30^ Details of this SNP are shown in Table S1. Though the population as a whole was not in Hardy Weinberg equilibrium (HWE) for this SNP (this population is known to be endogamous), HWE was not violated in the population of women taking exogenous oestrogens. The minor allele frequency for the population was 0.014 (Table S1). There was not any substantial missingness. Subsequent analysis was done in Rstudio.^31^ Participants who were homozygous or heterozygous for FVL were pooled for analysis due to low numbers of homozygotes prescribed Oestrogen (only 1).

#### Medication data from primary care

Medication data was acquired via linkage with primary care prescribing records. Not all participants had linked prescribing data available. Participating primary care clinical commissioning groups (CCGs) included: Tower Hamlets, Barking, Havering and Redbridge, City and Hackney, Newham, and Waltham Forest. Medication data was available for 85% of the total female population with linked genotype and clinical data (20,048/23,711). Participants without linked medication data were excluded from our analysis. Analysis relating to oestrogen containing medication use was thus undertaken in this cohort of 20,048 women.

#### Exogenous oestrogen use

Use of oestrogen contained in oral combined contraceptives was extracted from primary care prescribing records, using only those prescriptions listed as ordinary (as compared with short term) prescriptions. The following brand name OCP medications were included to target oral combined contraception (OCP) use: Bimizza, Gedarel, Mercilon, Akizza, Femodetter, Millinette, Sunya, Cimizt, Marvelon, Dretine, Lucette, Yacella, Yasmin, Yiznell, Femodene, Katya, Levest, Microgynon, Ovranette, Rigevidon, Elevin, Maexeni, Cilique, Lizinna, Brevinor, Norimin, Norinyl, Zoely, Logynon, TriRegol, Synphase, Qlaira. The non-brand names for oestrogens contained in these OCPs were also included: ethinylestradiol and estradiol, including non-oral formulations. A cut off participant age was not used (due to lack of confirmation of each woman’s age at menopause and age at time of prescription), thus our population may include women using oestrogens as Hormone replacement therapy (HRT). As these oestrogens have been associated with VTE in the context of use as OCP or HRT, this is a valid approach. None of the branded HRT patch therapies, gels, or pessaries were included.

#### G&H curated phenotypes

This analysis used G&H curated phenotypes for the medical co-morbidities of interest. These phenotypes were created using previously described methods.^32^^,^^33^ SNOMED codes, ICD codes and Office of Population Censuses and Surveys (OPCS) codes were obtained from linkage with electronic health records (including NHS digital, Barts Health NHS trust, Bradford teaching hospitals and primary care CCGs).^32^^,^^33^ The first occurrence of diagnoses was identified using UK Biobank methods, with the addition of primary and secondary care SNOMED code data.^34^^,^^35^

Diabetes mellitus (DM) included E10; type 1 diabetes mellitus, E11; type 2 diabetes mellitus, E13; other specified diabetes mellitus, E14; unspecified diabetes mellitus. Dyslipidemia was defined by ICD 10 code E78. Obesity was defined by ICD10 code E66. Chronic Kidney Disease (CKD) was defined by ICD10 code N18. Hypertension (HTN) was defined by ICD 10 code I10.

Venous thromboembolic events were identified from the above phenotypes using the following ICD 10 codes: Pulmonary embolism (I26), Phlebitis and thrombophlebitis (I80), Portal vein thrombosis (I81), Other venous embolism and thrombosis (I82).

#### G&H curated principal components

The G&H study team has prior published work using principal component analyses and made these available as curated parameters in the G&H trusted research environment.^28^ The first 20 of these principal components were used for this analysis to control for the influence of population stratification.

### Quantification and statistical analysis

Fisher’s exact test was used for comparison of discrete baseline characteristics between groups, and two sample t-test was used to test for difference in mean value between the two groups for continuous variables. Fisher’s exact test was used to compare prevalence of VTE in sub-cohorts. Multivariable logistic regression was used to test for association between prevalence of VTE and oestrogens use, FVL allele presence, and prevalence of common medical co-morbidities in the female sub-cohort. The first model included each medical condition specified as a co-variate. The second model included multi-morbidity as a multilevel variable to assess risk associated with presence of 1, 2, 3 or 4 of the following conditions in the same participant: Obesity, hypertension, dyslipidaemia, chronic kidney disease. These four conditions were used as they are common conditions, often co-occur, and each were independently significantly associated with increased VTE prevalence in the first step multivariate logistic regression analysis described above. This was a cross sectional analysis as dates of events were not available.

## Data Availability

• All Genes & Health data can be accessed by application to the study access team https://www.genesandhealth.org/research/scientists-using-genes-health-scientific-research. • Genes&Health phenotype generating codes are openly available to researchers and signposted at https://www.genesandhealth.org/research/phenotypes-list-counts.
