# Supplementary material for: Factor V Leiden, estrogen, and multimorbidity association with venous thromboembolism in a British-South Asian cohort
Source: iScience. 2023 Sep 1;26(10):107795. doi: 10.1016/j.isci.2023.107795 (PMC10550715; doi:10.1016/j.isci.2023.107795)
Supplement: Document S1. Tables S1 and S2 [file mmc1.pdf]

iScience, Volume *26*

Supplemental information

Factor V Leiden, estrogen, and multimorbidity association with venous thromboembolism in a British-South Asian cohort

Emma F. Magavern, Genes & Health Research Team, Damian Smedley, and Mark J. Caulfield

Supplementary table 1 – variant characteristics defining Factor V Leiden, related to the STAR Methods

**Rs ID**	RS6025
**Chromosome location**	1:169549811 (GRCh38)
**Allele change**	C>T
**MAF (N=20,048)**	0.014
**Missingness (fractional) (N=20,048)**	0.001
**HWE in Female G&H cohort (N=20,048)**	0.002
**HWE in only women exposed to oestrogens (N=5970)**	0.6

Supplementary table 2 - Female cohort characteristics and VTE events. P values from fisher's exact test for discrete variables and t-test for continuous variables, related to table 2.

* *p* value <0.05,

** *p* value <0.001

**Characteristics and co-morbid conditions**	**Prevalence in all Women (N 20,048)**	**Prevalence in Women with FVL** (N 558)	**Prevalence in Women without FVL** (N 19,490)	**P value**
Diabetes mellitus	15% (3,068)	15% (82)	15% (2,986)	0.7
Obesity	22% (4,410)	27% (150)	22% (4,260)	0.006*
Primary Hypertension	16% (3,298)	16% (89)	16% (3,209)	0.8
Dyslipidaemia	16% (3,192)	17% (97)	16% (3,095)	0.3
Chronic Kidney Disease	4% (831)	4% (24)	4% (807)	0.8
Mean Age at enrolment (years)	39 years old (+/- 13.2)	39 years old (+/- 13.9)	39 years old (+/- 13.1)	0.5
**VTE events**				
Pulmonary embolism	0.5% (96)	0.9% (5)	0.5% (91)	0.2
Phlebitis and thrombophlebitis	1.7% (340)	3.9% (22)	1.6% (318)	0.0003**
Other venous embolism and thrombosis	0.1% (23)	0.4% (2)	0.1% (21)	0.1
Portal vein thrombosis	0.05% (10)	0% (0)	0.05% (10)	1
Total number of participants with VTE	2.2% (439)	4.7% (26)	2.1% (413)	0.0003**
Oestrogen prescription	30% (5,970)	27% (153)	30% (5,817)	0.2
